# Contrary Perceptions of Environmental Health and the Governance of the Bucaramanga Metropolitan Area, Colombia

**DOI:** 10.3390/ijerph20196838

**Published:** 2023-09-27

**Authors:** Alexander Rojas, Douglas Molina-Orjuela, Laura Peña-Rodríguez, Andrea Hernández-Quirama, Mauricio Rojas-Betancur, Claudia Amaya-Castellanos, Laura A. Rodríguez-Villamizar, Alvaro J. Idrovo

**Affiliations:** 1 Public Health Department, School of Medicine, Universidad Industrial de Santander, Santander 680002, Colombia; d6901715@unimilitar.edu.co (A.R.); ciamaya@uis.edu.co (C.A.-C.); laurovi@uis.edu.co (L.A.R.-V.); 2 GRIALI Research Group, Faculty of Political Sciences and International Relations, Pontificia Universidad Javeriana, Bogotá 110231, Colombia; douglasemolina@gmail.com; 3 School of Social Work, Universidad Industrial de Santander, Santander 680002, Colombia; laura.alejandrapero@gmail.com (L.P.-R.); ahernanq@uis.edu.co (A.H.-Q.); hmrojasb@uis.edu.co (M.R.-B.)

**Keywords:** environmental health, multilevel governance, air pollution, water pollution, health policy

## Abstract

The participation of civil society is essential for environmental health policies to be accepted. The objective of this study was to know the perceptions of government officials, members of civil society, and academics about environmental health problems and its governance in the Bucaramanga Metropolitan Area, Colombia. In the region, there is a strong citizens movement that defends the moorland ecosystem (páramo) as a source of drinking water for large-scale mining projects. A multi-method study was conducted, including the review of newspaper and scientific articles, a citizens survey, and interviews and focus groups with identified key stakeholders. The findings indicate that government officials prioritize their actions on issues related to air and water pollution and environmental education. In contrast, citizens prioritize water availability from the moorland ecosystem. There are some advances in the management of environmental health, mainly related to greater citizen awareness. Contrary perceptions among government officials, academics, and civil society prevent adequate prioritization of environmental health problems. Participation of civil society is absent in activities related to environmental governance. An ongoing citizens science experience engaging high school students and the academy can be the first meeting point with government officials in the pathway to improve the environmental governance in the territory. The participation of civil society in the environmental health governance must be enforced to broaden the issues of interest and prioritize the activities in short- and long-term policy planning.

## 1. Introduction

Environmental health in Colombia has been one of the most neglected areas of public health, despite its importance in a country with high biodiversity and natural wealth [1]. The country has a low number of experts in environmental health [2], and a high number of socio-ecological conflicts [3], with direct or indirect effects on human health, which are related to the systematic assassination of environmental leaders [4]. Adequate environmental health management requires the joint action of the government, civil society, and academia. Since the National Council for Economic and Social Policy’s (CONPES) 3550 document was promulgated in 2008 [5], the process of creating a comprehensive environmental health policy began in Colombia. While the greatest success has been the creation of the National Intersectoral Technical Commission for Environmental Health (CONASA) at the national level, and the Territorial Environmental Health Councils (COTSA) at the local level [6], the greatest obstacle has been the difficulty of intersectoral action, even though the National Public Health 10-year Plans include environmental health [7].

Public policy making is a complex process. An effective public policy requires the support of politicians, implementing organizations, and citizens, as well as the evaluation of its effectiveness and efficiency. So, public policies may be an important way of addressing social problems and promoting societal well-being [8]. Furthermore, public policies should be based on empirical analysis and seek to address market or political/social/economic system failures, promote economic growth, improve citizens’ quality of life, and promote equity. They include resource allocation, the implementation of regulations, the provision of public services, and the promotion of citizen participation. These collective decisions should be made by political stakeholders or groups of stakeholders, influenced by broader social and political factors, and reflect values, co-ordinate actions, and determine the use of policy instruments to fulfill general criteria such as quality of life and human rights. Together, they should form a complex network of interdependent decision makers in different areas of society [8,9].

The case of Bucaramanga, Colombia, has drawn national attention because in recent years, civil society has demonstrated massively in defense of the Santurbán moorland ecosystem (páramo), the main source of drinking water in the region [10]. Unlike other cases where mining was allowed which had a negative impact on the environment and health, triggering the process of claiming damages, in Bucaramanga, civil society mobilized against the permission for gold exploitation by a multinational mining company. However, this social fight has generated a socio-environmental conflict between the inhabitants of the páramo who conduct small-scale gold mining without mercury use, and the inhabitants of the city who consume the water coming from the mountain [11]. This fact motivated our research question: what are the perceptions of government officials, the members of civil society, and the academics about environmental health problems and the region’s governance in Bucaramanga Metropolitan Area, Colombia.

## 2. Materials and Methods

### 2.1. Bucaramanga Metropolitan Area (BMA) in Context

Bucaramanga, the capital of the department of Santander, is in the northeast of Colombia on a plateau of the eastern cordillera. The city is located at 959 m a.s.l. with a subtropical highland climate and annual mean temperature of 27 °C. The local economy depends mainly on activities in the education, health, and tourism sectors, as well as agribusiness, metal–mechanics, and the traditional production of footwear. Bucaramanga was known nationally in the past decade as “the city of the parks” for its conservation of green spaces across the city. The other municipalities of the BMA are Floridablanca, Piedecuesta, and Girón, and all four municipalities are closely related, since there is a permanent natural movement of population among them. In total, the BMA population in 2023 was estimated to be ~1,112,000 inhabitants.

Although it is not one of the most polluted cities in Colombia, the BMA has been the subject of scientific research related to water resources and wastewater treatments, most of it in connection with, or supported by, oil and gas production research led by the Colombian Petroleum Institute located in Piedecuesta. The earliest studies identified procedures for the oxygenation of wastewater and the development of water pollution indexes [12], and the more recent have evaluated the combination of rainwater harvesting systems (RWHS) and greywater reuse systems (GWRS) as alternatives for urban water management [13]. There have also been studies related to water quality and conditions related to gastrointestinal parasites and dengue prevention [14,15]. Bucaramanga also has an important tradition of studies on air pollution-related adverse effects. The first studies identified respiratory signs and symptoms among susceptible populations [16,17], and the more recent have reported a cluster of cases of childhood cancer, possibly associated with air emissions from an industry in the north of the city [18].

### 2.2. Study Approach

This study used a multi-method approach with a multilevel governance perspective, in which government and non-government stakeholders have a role in both the structure and processes related to environmental health management [19]. The methods included a review of newspaper and scientific articles, a citizens survey of perceptions of environmental health and governance, and interviews and focus groups with identified key stakeholders. More details of each method will be described below.

With the review of news and scientific articles, we sought to know the respondents’ perceptions of environmental health problems. The citizens survey sought to discover the level of involvement in environmental health problems in order to manage possible solutions. Finally, with the interviews and focus groups, the activities conducted by each stakeholder or group of stakeholders, the level of relationship with other stakeholders or groups of stakeholders, and the difficulties of having an effective environmental health governance in the AMB were identified.

### 2.3. Environmental Health Topics in Newspaper and Scientific Articles

A review of news in the main regional newspaper *Vanguardia* was conducted using the electronic digital archive search engine. Only news items from Bucaramanga, Floridablanca, Girón, or Piedecuesta between 2016 and April 2020 were considered. Only this newspaper was selected because it is the most traditional in the region and the one that historically addresses environmental issues on a regular basis. The news items were summarized and classified by subject, which allowed the identification of stakeholders and actions related to environmental health. Additionally, scientific articles on environmental health in the BMA were identified in the PubMed/Medline database. Only these databases were included, because in a previous study it was clear that most of the research in the region is found in them [20]. The search strategy included the words: Bucaramanga AND (water pollution OR air pollution OR odor OR mining OR noise). Articles that did not have results from the city or its surroundings, and those that did not directly address environmental health issues (especially some reviews, studies in other sites, or methodology articles), were excluded from the analysis.

### 2.4. Survey of Citizens’ Perceptions

The survey was conducted in times of a pandemic, so it was done virtually using the Google Drive platform, taking advantage of the educational media. Snowball sampling was conducted, beginning with individuals known to one of the researchers in four different areas of the BMA. To guarantee anonymity, only sex, age, educational level, knowledge of environmental policies and regulations (11 questions) and citizen experiences related to environmental government were asked (7 questions) and were included. Specifically, we inquired about the knowledge of policies and regulations related to environmental health: environmental protection, protection of water sources, air quality management, recycling and solid waste management, residential waste collection, management of waste generated at home, noise management, vehicle gas emission control, contamination control of commercial establishments, pro-environmental culture, and the encouragement of reforestation. The questions were based on findings from a previous study on environmental psychology in Colombia [21]. In addition, some experiences of citizen participation in activities related to environmental governance were investigated: knowledge about institutions in charge of environmental management, participation in the generation of environmental policy, opinion on environmental issues, information about the generation of environmental policies, and participation in the generation of environmental policies.

### 2.5. Interviews and Focus Groups with Key Stakeholders

**Figure 1:**
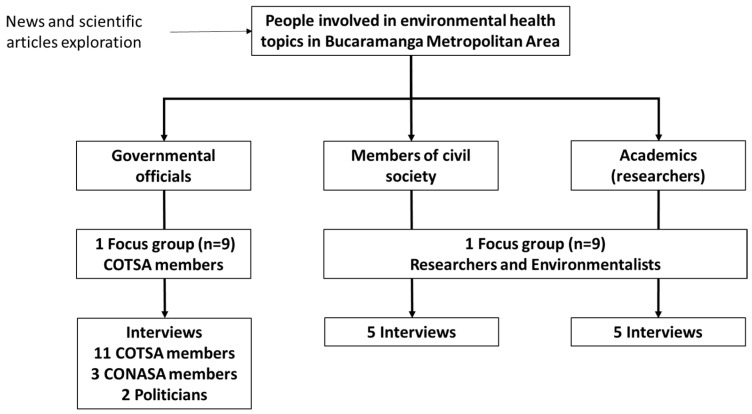
Participants in the focus groups and interviews included in this study.

The key stakeholders identified in the review of news and scientific articles allowed for a broad list of government, civil society, and academic stakeholders in environmental health issues (see Figure 1). First, two focus groups were conducted with government official members of COTSA and a group of researchers and environmentalists in which they were asked about their activities in environmental health, their relationship with other stakeholders, the perception of environmental health and territorial governance in the BMA, and their proposals to improve the governance of environmental health in the BMA. Government officials were clearer about their roles in environmental health governance, while this was not clear among civil society and academia members. Participation was mostly virtual because the focus groups and several interviews were conducted during the pandemic, between November 2020 and August 2022. In a second period, interviews with stakeholders were conducted to deepen the themes addressed in the focus groups. Some of the interviewees participated in the focus groups.

For the analysis of the information, the following categories were defined a priori: relations between social stakeholders, perception of environmental health problems in the territory, governance processes of environmental health in the territory, COTSA as a governance mechanism, perception of problems of the COTSA, and proposals for revitalization of COTSA. All the interviews were transcribed and later categorized with the support of Atlas.ti v7.0 software package.

### 2.6. Ethical Considerations

The study followed the guidelines for research with people as defined in Colombian regulations and the Declaration of Helsinki, and it was endorsed by the Scientific Research Ethics Committee of the Universidad Industrial de Santander. Specifically, the participants gave their written informed consent to participate and allow the recording of the meetings.

## 3. Results

### 3.1. Findings from Newspaper and Scientific Articles

A summary of the findings in journalistic notes is presented in Table 1. The most frequent news items related to environmental health in the BMA were related to offensive odors, followed by noise and air pollution; in Bucaramanga, the noise related with discos, bars, constructions, and automobile traffic were the most frequent. Offensive odors were related to sewage problems, a consequence of ~89% of sewage being discharged into the Río de Oro without treatment, landfill that exceeded its storage capacity, and industries dedicated to the manufacture of animal feed. Scientific articles in PubMed Medline addressed the issues of air pollution (*n* = 25), mining (*n* = 6), water pollution (*n* = 2), and noise and odor (*n* = 0).

The main stakeholders identified in the government were: the Ministerio de Salud y Protección Social, the Ministerio de Ambiente y Desarrollo Sustentable, the Corporación Autónoma Regional para la Defensa de la Meseta de Bucaramanga, the Área Metropolitana de Bucaramanga, the Secretaria de Salud Ambiental de Santander, the Secretaria de Salud y Ambiente de Bucaramanga, the Secretaria de Salud de Girón, the Secretaria de Salud de Floridablanca, the Secretaria de Salud de Piedecuesta, and the regional delegations of the Instituto Colombiano Agropecuario and the Instituto Nacional de Vigilancia de Medicamentos y Alimentos. In the academia, the main stakeholders identified were: the Universidad Santo Tomás de Aquino, the Universidad Pontificia Bolivariana, and the Universidad Industrial de Santander.

The pro-environmental actions that are conducted have well differentiated stakeholders. Odor annoyance is a matter for decision makers, since the interventions required are sanitary engineering which requires large investments. This includes improvements in wastewater treatment and reorganization of solid waste management, given that the capacity of the sanitary landfill has reached its maximum. The actions of civil society to clean up streams correspond to environmental awareness activities. Noise is an issue that affects civil society, which confronts it with complaints and legal demands. Air pollution is of interest to the academy and the environmental authority, and its main action has been to disseminate the findings of scientific research and, in recent years, to define an air quality management plan, respectively.

### 3.2. Survey of Citizens’ Perceptions

The survey participants were mostly women. By age, the women were between 27 and 59 years old (64.4%), followed by ≥60 years old (23.7%); among men, the majority were between 27 and 59 years old (61.5%) followed by the group between 18 and 26 years old (23.1%). The educational level was high, with the majority having technical or university studies (88.8%) or secondary studies (11.2%), which is evidence that the survey was answered by a non-representative sample characterized by respondents having a greater interest in environmental issues. The answers about knowledge of policies and regulations and their citizen experience in environmental governance are summarized in Table 2.

### 3.3. Interviews and Focus Groups with Key Stakeholders

The stakeholders were mostly professionals from various disciplines, among which environmental and agro-industrial engineering, veterinary medicine, medicine, chemistry, and law were more frequent. It was noteworthy that stakeholders from academia and civil society had more work and research experience in environmental health (generally >10 years). None of the government stakeholders interviewed had training in public health, epidemiology, or environmental health.

Participants agreed that there are no good relationships between the stakeholders, although there are exceptions (see Table 3). The environmental authorities are only aware of activities in the health sector when the health authority provides a report. In addition, co-ordination among the environmental authorities is also difficult, since one is on a departmental level, another from the Area Metropolitana de Bucaramanga, and another from the rest of the municipalities. This, which is a problem of a political nature, more serious in the Area Metropolitana de Bucaramanga, is complicated by the fact that there are some government officials who do not like to interact with other officials, focusing exclusively on their own duties. This same situation is repeated when dealing with information, where dialogue seems to be limited. The information is obtained upon direct request between the stakeholders. It is not presented as a dynamic process, of frequent exchange, but is mediated by the need and priority in the face of an environmental problem. On the contrary, in terms of research, alliances between institutions and academia are positively identified, even some of them long-standing, to deepen and broaden the sphere of knowledge on environmental issues of interest and/or respond to problems that require attention. In general, all the informants agree that joint action by the government, academia, and civil society is required to achieve positive effects on environmental health in the territory. For government officials, this means going beyond their mission competencies, recognizing the importance of working with other institutions and expending greater effort due to the time required and respect for understanding different ways of perceiving problems.

When inquiring about the concept of environmental health, it was noteworthy that academia and a small proportion of government officials provided a broad and complex definition including ecosystems, animals, and humans. Most government officials understand environmental health to be limited to those elements in the environment that affect human health. These differences in the concept of environmental health have deep implications for governance since they deny the possibility of understanding it as an intersectoral meeting point. Among the informants, the problem of odor annoyance is the most important, followed by air pollution and noise. A few mentioned as problems the use of toxins in homes such as asbestos in construction and pesticides in food, mishandling of pets, inadequate handling of farmyard animals, the presence of wildlife in urban areas, the COVID-19 pandemic, insufficient mobility infrastructure, global warming, and food safety. One informant also pointed out Venezuelan immigration, poverty, and corruption are environmental health problems.

Participants identified three environmental health governance processes in the territory: water governance, air governance, and environmental health governance. Water governance is the most relevant process and is linked to the defense of the Páramo de Santurbán against large-scale gold mining. The Committee for the Defense of the Water of the Páramo de Santurbán emerged in 2010 from this process, an alliance in which academics, technicians, and lawyers have conducted organized work that has prevented the approval of the large-scale mining license for transnational mining companies. This process has broad citizen support, including from private companies. In relation to air governance, academics and officials from environmental institutions recognize the importance of air quality. Actions based on scientific research have allowed decisions, mainly in the Bucaramanga mayor’s office, to reduce pollution. The main measures have been the ban on car use for one day a week (“pico y placa”), “car-free days”, and incentives to use bicycles. Unfortunately, its impact has been very small in terms of reducing pollution. Finally, the environmental health governance shows progress and setbacks (see Table 4).

In 2013, the Secretaria Departamental de Salud of the Santander promoted the creation of the departmental COTSA. It currently comprises the Corporación Autónoma Regional para la Defensa de la Meseta de Bucaramanga, the Corporación Autónoma de Santander, the Area Metropolitana de Bucaramanga, the Secretaria de Educación Departamental, the Secretarias de Salud of the BMA, the Secretaria de Agricultura Departamental, the Secretaria de Transporte e Infraestructura Departamental, the Secretaria de Planeación Departamental, the regional delegations of the Instituto de Hidrología, the Meteorología y Estudios Ambientales, the Instituto Colombiano Agropecuario, the Instituto Nacional de Vigilancia de Medicamentos y Alimentos, and the Instituto Colombiano de Bienestar Familiar, and the Procuraduria Ambiental y Agraria of Santander. The COTSA has a presidency, which alternates between the institutions, and a technical secretary who is always assumed by the Secretaria de Planeación Departamental. Internally, it comprises work groups for water and basic sanitation, chemical safety, air quality, food quality and safety, vector-borne diseases, and healthy environments. Unfortunately, at the meetings, only the activities conducted by each entity are reported, but no joint actions are planned. Minutes of the meetings are kept, fulfilling the legal requirement to meet at least once a year, but the processes conducted by COTSA are not effective. It has not been possible to have a unified budget allocation among the institutions that manages to have a clear impact on the problems of contamination and environmental deterioration associated with adverse effects on health.

For some officials, the main problem is that Colombia does not have a comprehensive environmental health policy (PISA, in Spanish) that serves as a guideline for the intersectoral activities of the institutions. Other problems are related to the lack of a budget for its operation, the insufficient organization, the management and planning of COTSA, the high turnover of personnel, the lack of knowledge in environmental health topics, the corruption, the lack of commitment, and a culture that is reactive in the face of problems. The proposals of the participants to improve the operation of COTSA are that the direction be from the academy, the inclusion of new stakeholders such as representatives of civil society, the establishment of a joint intersectoral action plan, to increase the frequency of meetings, and to demand participation of undefined-term contract workers of government institutions.

On the other hand, in the BMA, there is a very interesting experience of citizen participation in air quality monitoring which has not had sufficient support and dissemination. This is the case of the *Environmental Citizen Monitoring Network* (*Red Ambiental Ciudadana de Monitoreo*—RACIMO, in Spanish). RACIMO is a network of low-cost automatic and autonomous weather stations located in six public and private schools and the Universidad Industrial de Santander´s main campus, which provides information on air quality. The reports are disseminated in a didactic way on social networks, so that citizens can find out about air quality and the associated risk of disease [22]. Recently, the schools included in the RACIMO network have been increasing in numbers.

## 4. Discussion

**Figure 2:**
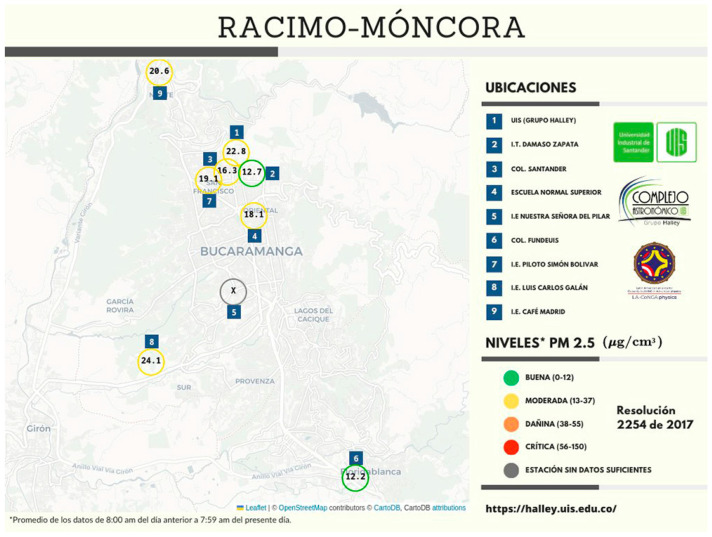
Infographic example of air quality in the BMA by the Environmental Citizen Monitoring Network (RACIMO). BUENA (0-12) represent GOOD (0-12); MODERADA (13-37) represent MODERATE (13-37); DAÑINA (38-55) represent HARMFUL (38-55); CRÍTICA (56-150) represent CRITICISM (56-150); ESTACIÓN SIN DATOS SUFICIENTES represent STATION WITHOUT SUFFICIENT DATA; Resolución 2254 de 2017 represent Resolution 2254 of 2017.

The main finding of this study was the identification of two contrary perceptions of environmental health problems in the BMA. While civil society emphasized that the threat of drinking water contaminated per mining is the main problem, government officials have prioritized air pollution by restoring the monitoring network coupled with monitoring of offensive odors (see Figure 2). Water sampling continues to be done routinely, and the Corporación Autónoma Regional Para la Defensa de la Meseta de Bucaramanga works on the recovery of water basins. Environmental education continues, especially with school children, while the search for a new sanitary landfill or the implementation of a new form of solid waste management has not had a solution. Academics consider that citizen participation in environmental health governance of the BMA is important, but their research interests focus on air pollution. This forces us to identify why the importance of air pollution and its effects on human health arises, which can be partially explained by the fact that water is visible to society while air is invisible, but also by the burden of disease studies and the financing of studies on the subject.

Studies of the burden of environmental disease in Colombia [23] have emphasized air pollution, unsafe water, sanitation and handwashing, occupational risks, and “other risks” because they have followed the international methodology of these type of studies [24] that describe the most representative environmental health problems in low- and high-income countries [25,26]. This has been criticized because it does not include other environmental exposures and adverse health effects which may be specific to some regions [27], such as exposure to environmental agents associated with neurotoxicity and endocrine disruption, among many others. Moreover, there is evidence that international and national funding for air pollution research has been prioritized [28] to better understand global problems such as climate change.

Prioritization of air quality as an environmental health policy over other health problems is common in large Colombian cities. For instance, in Bogotá, being the capital of the country, with the largest number of inhabitants and automobiles, with an important industrial sector, having the largest air monitoring network in the country, and having innovative experiences in mass urban transportation such as the Bus Rapid Transit System (Transmilenio) [29] and special routes for bicycle transportation (ciclo-rutas) [30], is an ideal place to research air quality issues. Another good example of contradiction between lay people and experts is Barranquilla, where the civil society perceives urban flooding, related to continuous dumping of solid waste into the sewer, as very important due to the damage, injuries, and deaths it causes during the rainy season [31].

When analyzing civil society, it is important to point out that the interested environmentalist groups present in the territory are small, and their actions for local impact have not been successful in managing a fluid dialogue with government officials and decision makers. This can be explained by (i) a lack of recognition of adverse effects associated with environmental exposures, (ii) lack of knowledge on reports generated by government institutions that indicate health problems due to environmental exposures, (iii) the perception of risk that arises from facts considered closer, such as the possibility of reducing the availability or quality of water, the possibility of chemical contamination by mining activities, offensive odors and noise, in the face of the invisible risk of air pollutants, (iv) lack of knowledge of the actions conducted by state institutions, control bodies, and the academy itself in relation to environmental health issues, and (v) the denial of participation in technical round-table discussions to talk about environmental health issues.

The RACIMO initiative [22] contributes to the construction of a change for social transformation supported by the Habermas triad, which includes technical, practical, and emancipatory interests. Students, while learning physics, analyze and appropriate social variables of participatory action research [32]. Although RACIMO data are valid and consistent with those of the government environmental monitoring network, according to an academic leader, there is no evidence of collaboration between citizens and government officials. They seem to perceive that their work can be replaced or audited by civil society. This can be understood since there is a high turnover of personnel in charge of environmental health, which has been related to governmental bureaucracy. Thus, there is evidence that not only is there a different prioritization of environmental health problems in the BMA, but also that citizen participation in issues that are considered specialized is minimal. In this sense, the RACIMO experience, which has a strong relationship between civil society and academia, can serve as point of integration with government officials. The RACIMO initiative should be seen as a strategy to broaden the air quality data generated by the official monitoring network, as the low-cost monitors offers complementary data [33]. Until now, the case of the BMA has been added to similar experiences where the construction and development of environmental public policies in Colombia has presented little or no participation from society [34,35].

Given that there are marked differences between men and women in relation to interest in environmental issues [36] and in the perception of risk in the face of environmental health problems [37], it is important to promote the participation of men and women among civil society, academia, and government officials. An analysis of environmental issues with a gender perspective allows for different visions of the problems, as well as proposing intervention strategies considering the particular needs and differential expectations between genders [38,39].

Our study has important limitations that must be considered in the interpretation of the findings. The study should be understood as descriptive and exploratory, with no search for causal associations. In order to advance in the improvement of environmental health governance in the territory of the BMA, it is not necessary to understand the causal associations. A complete evaluation of public policies in environmental health does require a more in-depth approach, but it is beyond the interests of this study. In fact, the political consequences of the study had repercussions in the mutual recognition of the stakeholders, the new training activities in environmental health, and recommendations for future activities in COTSA.

## 5. Conclusions

While for government officials the most important environmental health problem is air pollution-related adverse effects, which is supported by scientific studies by academics, for citizens, the main problems are offensive odors and drinking water quality, and availability from the Santurbán moorland. The latter is related to the possibility of open pit gold mining in high-altitude mountainous regions. The care of the banks of the water sources that pass through the city and environmental education are topics of shared interest. These different approaches can be understood with the difference in risk perception between lay people and experts, which is reinforced by the presence of national leadership of researchers in air quality and their health effects in the BMA. This study also identified that there is pending action from academia to educate the civil society about the risks of environmental exposures and the government officials in environmental health.

For environmental health governance to be truly effective, co-operation between government officials, citizens, and academics is crucial. The participation of civil society in the environmental health governance must be enforced in the BMA to broaden the issues of interest and prioritize the activities in short- and long-term policy planning. This will make it possible to make visible the problems that are not currently addressed, and to prioritize actions to improve the environment and prevent environmental diseases under the surveillance and support of citizens. The multilevel governance approach is fundamental to achieve this goal, since it will allow public policies to be oriented from the central level and actions in the territory to be the result of joint execution between decision makers, surveillance and control institutions, civil society, and academia.

## Figures and Tables

**Table 1:** Mean findings of news items in the *Vanguardia* newspaper (2016–2020) on environmental health problems in the Bucaramanga Metropolitan Area.

	Odor Annoyance and Water Pollution	Noise	Air Pollution
Year of publication			
2016	92	52	37
2017	105	86	45
2018	117	66	40
2019	26	46	42
2020 (until April)	4	6	23
Actions to solve problems (examples)			
Stakeholders	Environmental agency, mayor’s offices, and civil society	Civil society	Universities and Corporación Autónoma Regional para la Defensa de la Meseta de Bucaramanga
Specific action	Cleaning of water sources Creek cleaning and environmental education	Complaints, mobilizations, and lawsuits	Scientific studies and dissemination of results
	Debate on landfill issues		

Source: Database of news published in the regional newspaper *Vanguardia*.

**Table 2:** Findings obtained in the citizens’ survey in the Bucaramanga Metropolitan Area (*n* = 105).

Variable	Yes	No	No Response
Knowledge of policies and regulations			
Environmental protection	48.0	46.9	5.1
Protection of water sources	31.6	62.2	6.2
Air quality management	36.7	55.1	8.2
Recycling and solid waste management	62.2	36.7	1.1
Residential waste collection	56.1	37.8	6.1
Management of waste generated at home	53.1	43.9	3.0
Noise management	35.7	58.2	6.1
Vehicle gas emission control	40.8	52.0	7.2
Contamination control of commercial establishments	26.5	64.3	9.2
Pro-environmental culture	25.5	67.3	7.2
Encourage reforestation	22.4	70.4	7.2
Citizen experience in environmental governance			
You know institutions in charge of environmental management	72.4	23.5	4.1
Participation in the generation of environmental policy	10.2	87.8	2.0
You have been asked for your opinion on environmental issues	17.3	80.6	2.1
Has received information for the generation of environmental policies	18.4	79.6	2.0
Citizen participation in the generation of environmental policies is important	93.9	4.1	2.0
If you are invited, you would participate in the generation of environmental policies	88.8	4.1	7.1
Decisions on environmental management must be discussed and agreed between the government and citizens	98.0	1.0	1.0

Source: Database of survey of citizens’ perceptions.

**Table 3:** Examples of interaction between environmental health´s stakeholders in the Bucaramanga Metropolitan Area.

Control and surveillance	“When, for example, the Corporación Autónoma Regional para la Defensa de la Meseta de Bucaramanga requests an accompaniment to verify an odor problem with a farm, that does not happen every day. But when it happens, the Instituto Colombiano Agropecuario veterinarian goes to accompany them.” (Int-11) “The truth is, that I have not had very good dialogue with the environmental authorities; we have not worked very well, it is a sad reality.” (Int-5)
Information management	“What we have had to do is demand from the State entities, so we have requested information from the different health entities such as the Bucaramanga Municipal Health Secretariat and the Departmental Health Secretariat.” (Int-1) “Frequently we use, let’s say, official or governmental air information, for example, so I have contact with the environmental, local, national, and health authorities.” (Int-8)
Development of research in environmental health	“From co-ordination, we execute inter-administrative agreements with the academy, with the Universidad Pontificia Bolivariana, with the Universidad de Santander, Universidad Industrial de Santander, Universidad de Pamplona, Universidad Santo Tomás, with the Colombian Meteorological Service in which we generate environmental research projects, taking into account the environmental priorities that exist in the region. The Corporación Autónoma Regional para la Defensa de la Meseta de Bucaramanga has an environmental research plan, which has several strategic lines, and we have research projects that we need to develop and those priority research projects are the ones that we develop with the academy.” (Int-4) “With the Universidad Industrial de Santander, we have supported each other since 2007 in various phases of research on air quality and health, in different projects […] We already had the first meeting this month; the sites where the different systems to be measured have been chosen. Here PM_2.5_ will be measured, in different strategic sites in Bucaramanga, ozone will also be measured…” (Int-4)
Co-ordination of activities	“The municipalities (of the Area Metropolitana de Bucaramanga) have their own environmental health officers from their mayors’ offices, but the other 82 remaining municipalities that are category 4, 5, and 6, those are the responsibility of the Department.” (Int-5) “Here, at the Corporación Autónoma Regional para la Defensa de la Meseta de Bucaramanga, we only know how environmental health is being managed when the Ministry of Health issues the reports (…). There is no more direct communication, each institution does its part according to what corresponds to them.” (Int-4)

Source: interviews and focus groups.

**Table 4:** Progress and setbacks in environmental health governance in the Bucaramanga Metropolitan Area.

More pro-environmental awareness	“… the pressure that there is towards the rulers, commitment of the same civil society, in reducing contaminants, trying to reduce water consumption, generating minimum waste; in other words, everything, between the public and the private, civil society, educational institutions have also collaborated a lot, there is a lot of commitment in recent years; it has been seen that young people are more committed.” (Int-7)
Increased data availability	“(about air data) there is information that was not available before and had to be managed in a particular way, waiting for them to consolidate it; now there is information that is more freely available, others not so much but it can be requested and accessed…” (Int-15)
Less technological and workforce capacity	“With the Corporación Autónoma Regional para la Defensa de la Meseta de Bucaramanga, many years ago, we had a study group on air quality that we moved in (scientific) congresses. The Corporación Autónoma Regional para la Defensa de la Meseta de Bucaramanga managed the equipment of the air quality system well, but due to economic problems that network fell down. It was a national example because they had automatic equipment and they had professionals who could manage the network.” (Int-4)
Political disputes with technical consequences	“But here, too, each one does his own thing; look at the fight between the Area Metropolitana de Bucaramanga and the Corporación Autónoma Regional para la Defensa de la Meseta de Bucaramanga a few years ago; instead of working together, many times the authorities fight among themselves, so there is no such integration.” (Int-14)
Growth of environmental deterioration	“The results are not good, the processes have to be discussed (in conjunction) with the results. We are already in the 21st century and Bucaramanga treats only 10% of its wastewater, to give you an example. And the city grows, more wastewater is produced, more problems occur, deforestation increases because in Bucaramanga in the ’70s and ’80s it was much greener, but that is being lost.” (Int-2)

Source: interviews and focus groups.

## Data Availability

The data presented in this study are available on request from the corresponding author.
